# Identification of Six Novel *PTH1R* Mutations in Families with a History of Primary Failure of Tooth Eruption

**DOI:** 10.1371/journal.pone.0074601

**Published:** 2013-09-18

**Authors:** Lotte Risom, Line Christoffersen, Jette Daugaard-Jensen, Hanne Dahlgaard Hove, Henriette Skovgaard Andersen, Brage Storstein Andresen, Sven Kreiborg, Morten Duno

**Affiliations:** 1 Department of Clinical Genetics, University Hospital Copenhagen, Copenhagen, Denmark; 2 Centre for Rare Oral Diseases, University Hospital Copenhagen, Copenhagen, Denmark; 3 University of Southern Denmark, Odense, Denmark; 4 Department of Paediatric Dentistry and Clinical Genetics, University of Copenhagen, Copenhagen, Denmark; Oslo University Hospital, Norway

## Abstract

Primary Failure of tooth Eruption (PFE) is a non-syndromic disorder which can be caused by mutations in the parathyroid hormone receptor 1 gene (*PTH1R*). Traditionally, the disorder has been identified clinically based on post-emergent failure of eruption of permanent molars. However, patients with *PTH1R* mutations will not benefit from surgical and/or orthodontic treatment and it is therefore clinically important to establish whether a given failure of tooth eruption is caused by a *PTH1R* defect or not. We analyzed the *PTH1R* gene in six patients clinically diagnosed with PFE, all of which had undergone surgical and/or orthodontic interventions, and identified novel *PTH1R* mutations in all. Four of the six mutations were predicted to abolish correct mRNA maturation either through introduction of premature stop codons (c.947C>A and c.1082G>A), or by altering correct mRNA splicing (c.544-26_544-23del and c.989G>T). The latter was validated by transfection of minigenes. The six novel mutations expand the mutation spectrum for PFE from eight to 14 pathogenic mutations. Loss-of-function mutations in *PTH1R* are also associated with recessively inherited Blomstrand chondrodysplasia. We compiled all published *PTH1R* mutations and identified a mutational overlap between Blomstrand chondrodysplasia and PFE. The results suggest that a genetic approach to preclinical diagnosis will have important implication for surgical and orthodontic treatment of patients with failure of tooth eruption.

## Introduction

Primary Failure of Tooth Eruption (PFE, MIM #125350) is a non-syndromic disorder where the eruption of the posterior teeth ceases prematurely in children or adolescents, despite clearance by bone resorption of the eruption path. No mechanical obstruction for the eruption has been described and both the primary and permanent teeth may be affected [1]–[3]. Tooth eruption can be divided into five separate stages according to Marks et all [4], and it is the pre-occlusal (post-emergent) phase that is affected in PFE. The condition is variable both in terms of the number of affected teeth and the degree of symmetry [5]. The affected permanent teeth become ankylosed and application of orthodontic forces will have no effect [2].

The disorder can be caused by mutations in the parathyroid hormone receptor 1 gene (*PTH1R*, chromosome 3p21-p22.1, MIM #168468), and it is inherited in an autosomal dominant fashion with variable phenotypic expression but almost complete penetrance [6]. Traditionally, patients with tooth eruption failure often undergo surgical and/or orthodontic treatment [1]. However, patients with *PTH1R* mutations have no beneficial effect of such a treatment regime [7], [8]. It is therefore clinically important to establish whether a given arrest of tooth eruption has a genetic cause [8].

Mutations in *PTH1R* have also been associated with Jansen chondrodysplasia (MIM: #156400), Blomstrand chondrodysplasia (BOCD, MIM: #215045), Eiken (MIM: #600002), and Ollier (MIM: #166000) disease. Thereby, PFE is the fifth known clinical entity associated with mutations in *PTH1R.* All five disorders are characterized by various defects in skeletal development. The genetic basis for the various diseases appears, however, to be distinct [9]–[14]. Jansen chondrodysplasia is caused by constitutive activating mutations and is dominantly inherited, whereas Blomstrand chondrodysplasia is recessively inherited, caused by loss-of-function mutations. Only a single homozygous mutation has been associated with Eiken disease, and in rare cases with Ollier disease sporadic mutations in *PTH1R* have been found in cancer tissue.

As the association between mutations in *PTH1R* and PFE is a recent discovery, the actual frequency of the disorder among patients with eruption failure is likely to be underestimated. This is reflected by the fact that only eight different mutations until now have been identified in PFE patients [6], [7], [15]. These mutations are all predicted to result in premature proteolytic degradation of the precursor protein or to impair correct mRNA maturation/translation. Thus, the likely cause of PFE is haploinsufficiency of *PTH1R*. This implies that carriers of Blomstrand chondrodysplasia should be genetically predisposed to PFE. However, no such connection has been reported, yet.

We have analyzed six patients with clinical presentation of familial PFE and identified six novel mutations in *PTH1R.* All identified mutations were found in multiple affected individuals within the family. Two presumed splice variants were validated using a *PTH1R* minigene. By compiling known mutations in *PTH1R* we identified mutational overlap between PFE and Blomstrand chondrodysplasia.

## Materials and Methods

Patients with a clinical diagnosis of familial PFE, where at least two members of the family were affected, were recruited from Resource Centre for Rare Oral Diseases and Dept. of Clinical Genetics, both Copenhagen University Hospital.

Informed consent was obtained from every adult participant or from a legal parent in case of minors according to the recommendations of the declaration of Helsinki. All patients were offered and underwent genetic counseling. One individual had counseling by phone due to long geographic distance at own wish. The analysis performed in this study was done in a clinical hospital setting and is a general hospital service provided to interested patients in line with other usual hospital services (national Danish medical legislation LBK nr 913 of 13/07/2010). According to Danish law, such retrospective analysis does not require consideration by an Ethics committee. In accordance with Danish health law, informed, written consent for publication was obtained from the participants, and procedures were confirmed with the department of Staff and Law at the University Hospital Copenhagen.

**Figure 1:**
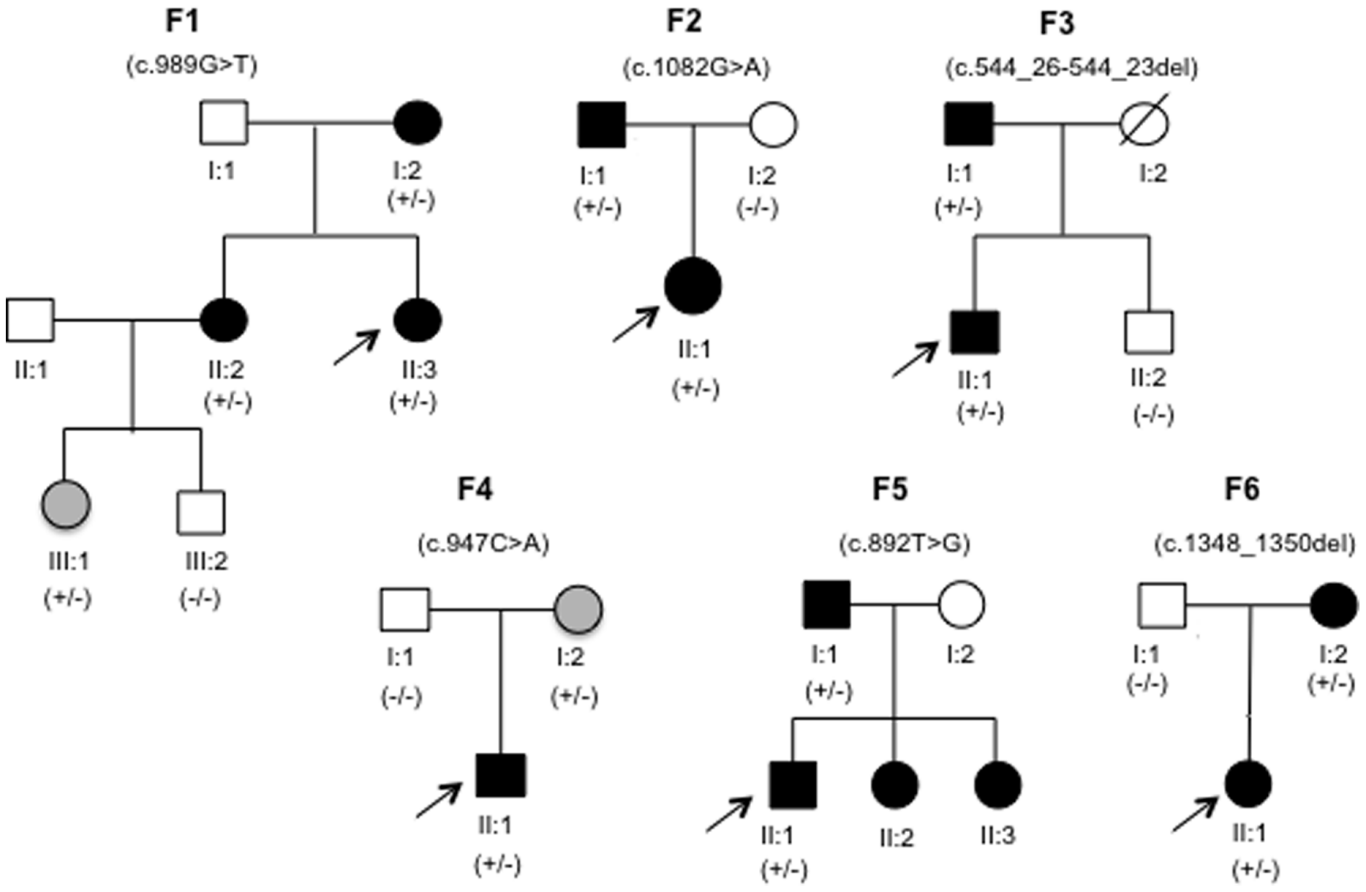
Pedigree diagrams of family F1–F6. Black symbols indicate clinically affected family members, open symbols indicate unaffected, and grey symbols indicate affected but with no clinical assessment/diagnosis. Arrow specifies the proband of the family. +/− denotes heterozygote presence of the familial mutation, whereas −/− denote absence. Not all family members were tested. Family 1 (F1) consisted of 7 members, 5 of which participated in the study. Families F2, F3, F4 and F6 consisted each of three members of which all participated. In family F5, two members participated and two additional members (F5; II:2 and F5; II:3) had clinically been diagnosed with PFE prior to the genetic study but declined genetic conformation.

Pedigrees were constructed based on clinical information and interviews of the families (Fig. 1) and blood samples or saliva mucosa were collected from one or several individuals from each family.

### Clinical Analysis

**Figure 2:**
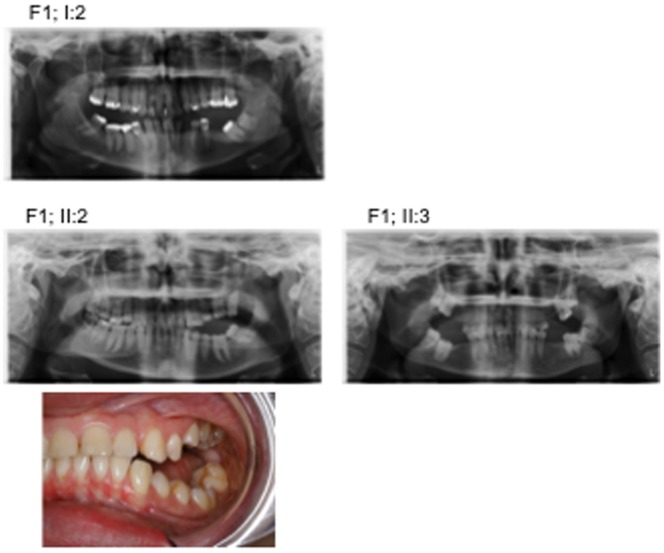
Orthopantomograms of three members from family F1. The pictures illustrate the variable phenotypic expression of the disorder in relation to symmetry and degree of affected teeth. F1; I:2 and F2; II:3 had affected teeth surgically removed, while F1; II:2 also had undergone unsuccessful orthodontic treatment. F1 II,2 is a 20 year old women showing primary failure of eruption (PFE) of maxillary and mandibulary premolars and molars. At age 16 the second molars in the left side of the maxilla and the mandible (27,37) were removed. The following orthodontic treatment was not succesfull. A new treatment plan including distraction osteogenesis of the posterior alveolar segment in the maxilla has been offered to the patient. The treatment has not yet been performed.

Clinical examination and orthopantomograms were used to assess the dental phenotype and the localization of the eruption problems (Fig. 2 and Table 1). All probands had undergone surgical and/or orthodontic treatment. In family F1 the proband (F1; II:3) had all four permanent first molars surgically removed and no further treatment, while her sister (F1; II:2) had undergone unsuccessful orthodontic treatment (Table 1).

**Table 1:** Characterization of Family Members with PTH1R Mutations.

					Affected Teeth																			
					Upper Right					Upper left					Lower left					Lower right				
Family ID	Sex	Age	PedigreePosition	Nucleotide Change	18	17	16	15	14	24	25	26	27	28	38	37	36	35	34	44	45	46	47	48
F1	F	58	I:2	c.989G>T	x	▪	▪	•	•	•	•	▪	▪	x	▪	▪	xx	•	•	•	▪	▪	xx	▪
	F	29	II:2	c.989G>T	•	•	•	▪	•	▪	▪	▪	xx	▪	▪	xx	▪	▪	▪	•	•	•	•	•
	F	23	II:3	c.989G>T	▪	▪	xx	•	•	•	▪	xx	▪	▪	▪	▪	xx	•	•	•	•	xx	▪	▪
	F	7	III:1	c.989G>T			•					•					•					•		
F2	M	56	I:1	c.1082G>A	No clinical observation																			
	F	28	II:1	c.1082G>A	▪	▪	xx	▪	▪	▪	▪	▪	▪	▪	▪	•	•	•	•	•	•	•	•	▪
F3	M	58	I:1	c.544-26_544-23del	▪	▪	•	x	•	•	x	•	•	▪	x	x	•	•	•	•	•	xx	xx	▪
	M	20	II:1	c.544-26_544-23del	▪	•	xx	•	•	•	•	xx	•	▪	x	▪	xx	•	•	•	•	xx	▪	x
F4	F	56	I:2	c.947C>A	No clinical observation																			
	M	27	II:1	c.947C>A	•	•	▪	•	•	•	•	•	•	•	▪	xx	xx	•	•	•	•	xx	▪	▪
F5	M	46	I:1	c.892T>G	x	•	•	•	•	•	•	•	▪	▪	x	▪	▪	•	•	•	•	•	•	x
	M	15	II:1	c.892T>G	▪	•	▪	▪	▪	▪	▪	▪	•	▪	▪	▪	▪	▪	▪	▪	▪	▪	▪	▪
	F	17	II:2	c.892T>G	x	▪	▪	▪	▪	•	•	▪	▪	x	•	•	▪	•	•	▪	▪	▪	▪	▪
	F	5	II:3	c.892T>G	No permanent teeth erupted, maxillary and mandibular primary molars affected																			
F6	F	57	I:1	c.1348_1350del	▪	x	▪	•	•	•	•	▪	x	x	x	▪	▪	▪	•	•	▪	xx	xx	▪
	F	22	II:1	c.1348_1350del	▪	▪	xx	▪	▪	•	▪	xx	▪	•	x	▪	xx	▪	•	•	•	•	•	x

Tooth present (•); not present (x); affected (▪); affected but extracted (xx). No entry denotes no information obtained.

### Genetic Analysis

DNA was isolated from a 6–10 ml EDTA blood sample using a standard desalting procedure, or from saliva mucosa (sample F4; I:1 and I:2) according to the manufacturer’s description (Oragene, DNAgenotek, Ontario, Canada). PCR primers were designed using the ExonPrimer program (http://ihg2.helmholtz-muenchen.de), and tailed with M13 sequences (see supplement Table S1). PCR amplification of the 16 exons was performed using the GoTaq PCR core systems I (Promega, Madison, Wisconsin) or the GC-rich PCR system (exon 3 and 9) (Roche, Hvidovre, Denmark). PCR products were purified on a NucleoFast96PCR plate (Machery-Nagel, Düren, Germany), directly sequenced on both strands using BigDyeTerminator V1.1, and subsequently resolved on an ABI3130 (Applied Biosystems, Foster City, USA). Identified variations were confirmed in a new PCR and sequencing reaction. NM_000316.2 was used as reference sequence.

### Minigene Reporter Assays

The two putative splice mutations c.544-26_544-23del (ID: F3) and c.989G>T (ID: F1), were analyzed by a minigene reporter assay [16]. Briefly, three minigenes where constructed each encompassing exon 7 to 12 harboring the putative splice mutations or a wild type (WT) sequence (Genscript, Piscataway, NJ). HEPG2 cells were cultured in RPMI1640 (Lonza, DK) supplemented with 10% fetal bovine serum (FBS; Sigma F7524), 2 mM glutamine (Gibco, Invitrogen, Nærum, Denmark), 100 U/ml penicillin/streptomycin at 37°C and 5% CO_2_. Prior to transfection, cells were grown to 50% confluence in six well plates and then transfected with WT or mutant constructs using Fugene6 according to the manufactory instructions (Roche, Hvidovre, Denmark). Cells were harvested 48 hours post transfection, and total RNA was isolated using Isol (5 PRIME, AH Diagnostics, Århus, Denmark) and reverse transcribed to cDNA (SuperScript VILO™ cDNA Synthesis Kit (Invitrogen, Nærum, Denmark). Subsequent PCR was performed using the primer pairs: vector primer T7-EXT (5′-attaatacgactcactataggg-3′) and PTH1R-cDNAex10R (5′-gttggtggccaggaagtaaa-3′) for c.544-26_544-23del variation or PTH1R-cDNAex10F (5′-tctcagagaagaagtacctgt-3′) and vector primer BGHREV (5′-aactgaaaggcacagtcgaggctg-3′) for c.989G>T variation. PCR products were analyzed by gel electrophoresis, extracted directly and purified for subsequent sequencing.

### In silico Analysis

Sequence data were initially inspected by eye and Mutation Surveyor V3.3. Unknown sequence variants were evaluated by SIFT, PolyPhen2, and AlignGVGD. Variants suspected to affect correct splicing were evaluated for splice site strength or branch point prediction based on 5 different algorithms (SpliceSiteFinder, MaxEntScan, NNSPLICE, GeneSplicer, Human Splicing Finder) through the bioinformatics tools of the Alamut software v.1.5 (Interactive Biosoftware, http://www.interactive-biosoftware.com/alamut.html).

## Results

All six pedigrees were compatible with autosomal dominant inheritance (see Fig. 1).

The entire coding and exon flanking sequence of *PTH1R* was sequenced in six probands with a clinical diagnosis of familial PFE. A heterozygous pathogenic variant was identified in all six cases (Table 2, for fluorograms see supplement figure S1). All six mutations were absent from the dbSNP, HGMD (subscription access) and the ESP databases, and have to our knowledge not been described previously. *In silico* analyses proposed a possible pathogenic effect of all variants. Extended familial analysis verified that all the novel mutations segregated with the PFE phenotype (Fig. 1).

**Table 2:** Summary of mutations in Blomstrand chondrodysplasia and PFE patients.

Disease	Nucleotide change		Expected protein change	Reference
*PFE*				
		c.356C>T	p.Pro119Leu	Yamaguchi et al. [15]
		^¤^c.395C>T	^¤^p.Pro132Leu	Yamaguchi et al. [1]
		c.439C>T	p.Arg147Cys	Yamaguchi et al. [15]
		c.463G>Tc.543+1G>A	p.Glu155*p.Glu182Valfs*20	Decker et al. [6]Decker et al. [6]
		c.544-26_544-23del	p.Glu182Alafs*38	This study
		c.892T>G	p.Trp298Gly	This study
		c.947C>A	p.Ser316*	This study
		c.989G>T	p.Gly330Val/altered splicing	This study
		c.1050-3C>G	p.Cys351Serfs*133	Decker et al. [6]
		c.1082G>A	p.Trp361*	This study
		#c.1148G>A	#p.Arg383Gln	Yamaguchi et al. [15]
		c.1348_1350del	p.Phe450del	This study
		c.1354-1G>A	p.Gly452_Glu465del	Frazier-Bowers et al. [7]
*Blomstrand chondrodysplasia*				
	c.1093delG		p.Val365Cysfs*141	Karperien et al. [25]
	c.310C>T		p.Arg104*	Hoogendam et al. [24]
	^¤^c.395C>T		^¤^p.Pro132Leu	Karaplis et al. [10], Zhang et al. [11]
	c.1049+27C>T		p.Cys351*	Hoogendam et al. [24]
	#c.1148G>A		#p.Leu373_Arg383del	Jobert et al. [9]

¤# Mutations found in both PFE and Blomstrand chondrodysplasia patients.

# c.1148G>A was initially suspected a classic missense mutation, but was later shown to affect correct mRNA splicing.

In families F2 and F4, two nonsense mutations, c.1082G>A (p.Trp361*) and c.947C>A (p.Ser316*), were identified. The two mutations are located in exons 10 and 12, respectively, and are both expected to activate the nonsense mediated mRNA decay pathway that selectively degrades mRNAs harbouring premature termination codons [17].

A 3bp deletion (c.1348_1350del) was identified in family F6, predicted to result in a deletion of p.Phe450. Phenylalanine in this position is highly conserved across different species and together with the clear phenotypic presentation and segregation we assume the deletion to be pathogenic.

Two variations, c.892T>G and c.989G>T, were predicted missense mutations. The c.892T>G (family F5) changes the highly conserved tryptophan (Trp) at position 298 to the much smaller amino acid glycine (Gly), p.Trp298Gly. Although both amino acids are hydrophobic the physicochemical difference between Trp and Gly is quite large, underscoring a pathogenic effect. The c.989G>T mutation (family F1) was initially predicted to result in p.Gly330Val. However, as it changes the first nucleotide of exon 11 we speculated whether c.989G>T might instead disrupt the 3′ splice-site of exon 11 and thus lead to aberrant splicing rather than changing the protein sequence.

In family F3, a second putative splice mutation was identified. This 4 bp deletion, c.544-26_544-23del, is predicted to disrupt the correct branch-point of intron 7 as it deletes part of the putative branch point sequence including the canonical A nucleotide of the YUN**A**Y consensus branch point sequence [18]. It is therefore likely, that this deletion will cause aberrant splicing.

**Figure 3:**
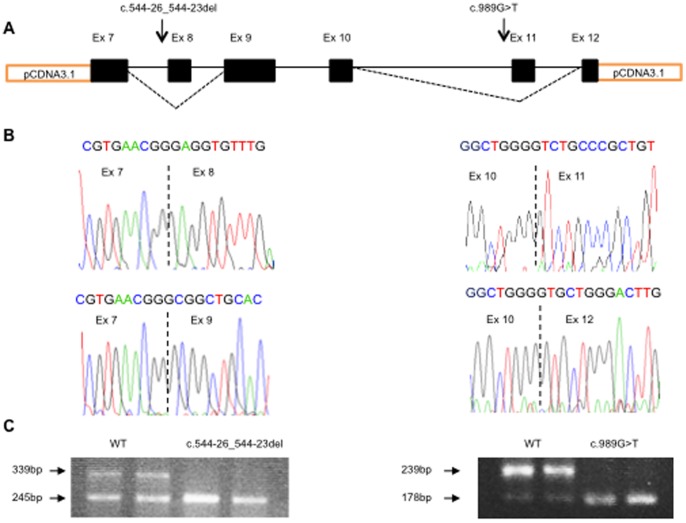
Functional analysis. Results of functional studies of two putative *PTH1R* splice mutations by minigene reporter analysis. (A) Schematic location of the two putative splice mutations and the observed exon skipping, indicated by dotted lines. The two mutations are for graphical reasons depicted on the same figure, but are obviously located on separate constructs (B). Sequencing results of normal cDNA minigene transcript and of cDNA from the c.544-26_544-23 (ID: F3) and c.989G>T (ID: F1) minigene transcripts, demonstrating complete skipping of exon 8 and exon 11, respectively. (C) Both normal and irregular splicing is observed for the normal allele in both analyses indicating that native exon 8 and 11 have weak 3′-splice sites. The putative branch point mutation c.544-26_544-23 results in complete skipping of exon 8 whereas the c.989G>T splice site mutation results in complete skipping of exon 11.

In order to investigate the effect of the two potential splice mutations, we constructed minigenes expressing exon 7 to 12 harboring the two mutations, transfected HEPG2 cells and performed cDNA analysis. The minigene assay confirmed that both c.989G>T and c.544-26_544-23del caused aberrant splicing (Fig. 3). The c.989G>T mutation caused complete exon 11 skipping which is deleterious. Apart from the complete skipping of exon 8 due to c.544-26_544-23del, we also observed a substantial skipping of the wild type construct. This indicates that native exon 8 is difficult to splice, probably due to the rather weak 3′ splice site. The Burge 3′ splice site score of exon 8 is 7.92 indicating a relative weak 3′ splice site [19]. Deletion of the branch point sequence caused complete exon 8 skipping, in full agreement with a deleterious effect of c.544-26_544-23del.

By comparing published mutations found in Blomstrand chondrodysplasia and PFE patients (Table 2), we have uncovered the c.1148G>A mutation, to be identified in both diseases [9], [15].

## Discussion

Tooth eruption disorders of posterior permanent teeth are complex and poorly understood conditions, which can be part of a syndrome or presented as a single clinical entity. The latter is referred to as “primary failure of eruption” (PFE) [1] and has an estimated prevalence of approx. 1/2000 [20].

Our findings are in agreement with previous studies showing variability in the number of affected teeth and the degree of symmetry both within and between the families [5]. In all 14 affected cases the eruption problem affected the pre-occlusal stage of eruption which is in agreement with Profitt and Vig [1]. About 25% of patients clinically diagnosed with PFE display a dominant family history of the condition indicating genetic heterogeneity [3]. Recently, mutations in *PTH1R* were shown to be a genetic cause of PFE, but only eight patients have been genetically verified, so far (Table 2). As PFE patients with *PTH1R* mutations do not benefit from surgical and/or orthodontic treatment, it is essential to investigate for *PTH1R* mutations prior to treatment planning [5].

We investigated six families with a clinical diagnosis of familial PFE and where at least two family members were affected, and each family was shown to harbor a novel mutation in *PTH1R*. Thus, our study expands the mutation spectrum for PFE from eight to 14 pathogenic mutations. All six probands had undergone unsuccessful surgical and/or orthodontic treatment before the causal relationship between PFE and *PTH1R* mutations had been established. Screening for *PTH1R* mutations in patients with PFE could therefore significantly reduce the need for surgical and/or orthodontic intervention. All patients were offered and underwent genetic counseling. Extended familial analyses verified that the identified mutations all segregated with the clinical phenotype. In family F1, the c.989G>T mutation was identified in the child (F1; III:1), but at the age of 7 years she was not clinically affected by PFE.

In family F2, the father (F2; I:1) was unavailable for clinical examination, but participated with an EDTA blood sample. Clinical information was available from an early surgical intervention due to failure of eruption of molar teeth. The mother of the proband in family F4 (F4; I:2) was counseled by phone only, and rejected dental examination due to anxiety. However, she did confirm “problems with her teeth” in agreement with the heterozygous presence of a non-sense mutation (c.947C>A, p.Ser316*).

In family F5, the proband and his father participated, but two sisters (F5; II:2 and F5; II:3) were not able to participate. However, clinical photos and orthopanthomograms were made available, and both sisters were clinically diagnosed as positive with PFE.

The putative splice mutation c.989G>T identified in family F1 affects the first base in exon 11. The guanine nucleotide (G) at this position is often crucial for correct splicing of adjacent exons [21]. In the 3′ splice site of intron 11 preceding c.989G>T there are only 10 uninterupted pyrimidines. Fu and co-workers suggest that at least 10–15 uninterupted pyrimidines are needed in order to tolerate a mutation of the initial G of an exon [21]. The wild type 3′ splice site has an arbitrary efficiency score of 12.82 which is reduced to 10.61 by c.989G>T in agreement with a weakening of the 3′ splice site of exon 11 [19]. Results from the mini-gene reports assay confirmed that the mutation result in a skipping of exon 11 rather than being translated into the missense mutation p.Gly330Val (Fig. 3). Skipping of exon 11 theoretically result in an *in-frame* transcript however; a removal of 20 amino acids located centrally in the protein is most likely detrimental for correct protein function.

Validation of the second putative splice mutation, c.544-26_544-23del, identified in family F3, indicated that the native 3′ splice site of exon 8 is rather weak allowing some degree of illegitimate splicing, whereas the four base pair deletion fully abolished correct splicing (Fig. 3). Skipping of the entire exon 8 creates a frame shift (p.Glu182Alafs*38) introducing a premature stop codon 37 bases downstream in the mRNA transcript and will most likely be targeted for NMD.

Thus, four of the six identified mutations are predicted to terminate correct mRNA maturation/translation in agreement with haploinsufficiency of *PTH1R* as the genetic basis of PFE.

*PTH1R* encodes a G protein coupled receptor for parathyroid hormone (PTH) and parathyroid hormone related peptide (PTHrP) [22]. A key function of the receptor is to regulate the calcium metabolism in connection with skeletal development [23] and *PTH1R* mutations have been implicated in rare diseases such as Jansen chondrodysplasia and Blomstrand chondrodysplasia. The latter is recessively inherited and caused by loss-of-function mutations [10], [11], [24], [25]. *Pth1r^−/−^* mice have comparable phenotypic characteristics to Blomstrand patients [26] and studies of Blomstrand fetuses have shown anomalies of deciduous teeth [27]. It is therefore surprising, that no heterozygous carriers of Blomstrand chondrodysplasia have been reported with symptoms of PFE. It has been suggested that carriers of Blomstrand chondrodysplasia could have unreported tooth eruption disturbances due to an incomplete medical history [6], or that absence of PFE in obligate carriers is due to difference in the mutation spectrum between PFE and Blomstrand chondrodysplasia patients. The latter suggestion is, however, contradicted by the recent identification of the c.395C>T mutation in a PFE patient [15] which originally was discovered in Blomstrand chondrodysplasia patients [9], [10]. Also the uncovering of the c.1148G>A mutation to be identified in both diseases [9], [15] contradict to the suggestion that the mutation spectrum is different between the two diseases. Lastly, four out of the five mutations known to be associated with Blomstrand chondrodysplasia are predicted to compromise correct mRNA function, much alike the mutations found in PFE patients. Altogether, this implies that carriers of Blomstrand chondrodysplasia are genetically predisposed to PFE.

Mutations in *PTH1R* causing PFE are thought to exhibit complete penetrance, although a variable phenotypic expression can be observed (see Table 1). Twelve of the 14 reported mutations are concordantly found in multiple affected individuals within the same family, whereas only two, c.395C>T and c.439C>T, have been discovered in apparent simplex patients. No attempt to investigate related family members were, however, reported [15]. As both mutations are predicted missense mutations (p.Pro132Leu and p.Arg147Cys), it could be speculated whether the resulting receptor might exhibit residual activity, thus leading to incomplete penetrance. One of the mutations, c.395C>T, has, however, been identified in a Blomstrand chondrodysplasia patient arguing against this assumption.

A genetic approach to preclinical diagnosis by identification of mutations in *PTH1R* makes it possible to perform segregation analysis of future affected relatives, ensuring an early diagnosis and, thereby, a correct treatment scheme. Together, the prevalence of PFE and number of patients displaying genetic heterogeneity, and the compiled results of Blomstrand chondrodysplasia and PFE mutations indicate that PFE caused by mutations in *PTH1R* is potentially underestimated.

## Supporting Information

Figure S1 **Flourograms.**
(TIFF)

Table S1 **List of primers.**
(DOCX)
