# Supplementary figures and images for: Identification of Six Novel PTH1R Mutations in Families with a History of Primary Failure of Tooth Eruption
Source: PLoS One. 2013 Sep 18;8(9):e74601. doi: 10.1371/journal.pone.0074601 (PMC3776825; doi:10.1371/journal.pone.0074601)

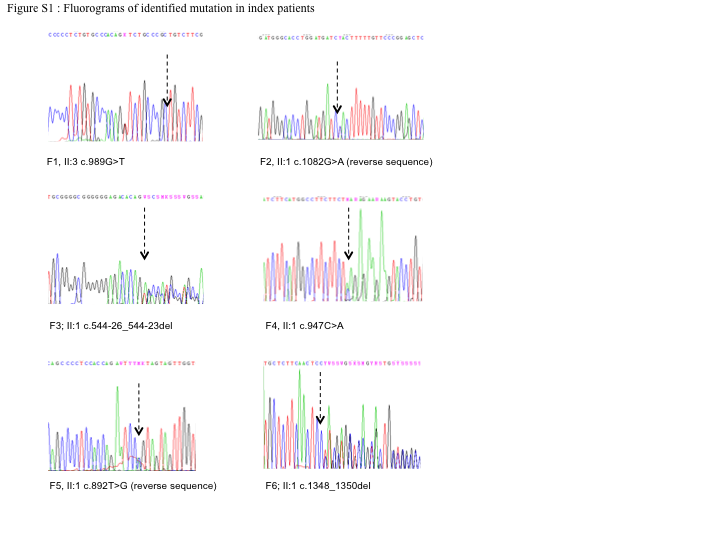

Supplement: Figure S1 — Flourograms. (TIFF) [file pone.0074601.s001.tiff]
