# Supplementary material for: Identification of Six Novel PTH1R Mutations in Families with a History of Primary Failure of Tooth Eruption
Source: PLoS One. 2013 Sep 18;8(9):e74601. doi: 10.1371/journal.pone.0074601 (PMC3776825; doi:10.1371/journal.pone.0074601)
Supplement: Table S1 — List of primers. (DOCX) [file pone.0074601.s002.docx]

**Table S1:** List of primers

| **Name** | **Exon** | **Primer direction** | **Length bp** | **Tm °C** | **Primer sequence** | **Product size/bp** | **PCR annealing temperature** | **PCR protocol** |
| --- | --- | --- | --- | --- | --- | --- | --- | --- |
| PTH1R_Ex1_M13_F  PTH1R_Ex1_M13_R | 1 | Fw  Rev | 35  38 | 57.66  60.54 | 5'-tgtaaaacgacggccagtGCCAGTGTAGGGCTTGG-3'  5'-caggaaacagctatgaccCCAGGGAAACCACAGACTTG-3' | 308 | 58°C | GoTaq PCR Core System I |
| PTH1R_Ex2_M13_F  PTH1R_Ex2_M13_R | 2 | Fw  Rev | 39  36 | 60.76  60.48 | 5'-tgtaaaacgacggccagtCCACCCTCGTGGAAACTAAAC-3'  5'-caggaaacagctatgaccGAAGCGAATGCATGAGGC-3' | 233 | 58°C | GoTaq PCR Core System I |
| PTH1R_Ex3_M13_F  PTH1R_Ex3_M13_R | 3 | Fw  Rev | 35  38 | 62.02  60.24 | 5'-tgtaaaacgacggccagtCTCCTTTGCGCTGCTCG-3'  5'-caggaaacagctatgaccTGCGTGCCTTAGACCTATCC-3' | 400 | 58°C | GC-rich PCR System |
| PTH1R_Ex4_M13_F  PTH1R_Ex4_M13_R | 4 | Fw  Rev | 36  37 | 60.72  60.47 | 5'-tgtaaaacgacggccagtCTCTGGGACCTGCTGCTG-3'  5'-caggaaacagctatgaccCTCTGTATCCTGGGTCCCG-3' | 279 | 58°C | GoTaq PCR Core System I |
| PTH1R_Ex5_M13_F  PTH1R_Ex5_M13_R | 5 | Fw  Rev | 38  39 | 58.72  60.88 | 5'-tgtaaaacgacggccagtTGGGTCTCCTGTTGTAGCAC-3'  5'-caggaaacagctatgaccGCTCACATCAGAGGGACAGTG-3' | 307 | 58°C | GoTaq PCR Core System I |
| PTH1R_Ex6 7_M13_F  PTH1R_Ex6 7_M13_R | 6+7 | Fw  Rev | 38  35 | 59.09  60.03 | 5'-tgtaaaacgacggccagtAATCATGGCCTTGACTCTCC-3'  5'-caggaaacagctatgaccGCTGGGTCAGGGGTCAC-3' | 503 | 58°C | GoTaq PCR Core System I |
| PTH1R_Ex8_M13_F  PTH1R_Ex8_M13_R | 8 | Fw  Rev | 38  37 | 59.99  60.10 | 5’-tgtaaaacgacggccagtCTGACCTTGACTCCTCCAGC-3’  5’-caggaaacagctatgaccGGACAGGAAGCTGGGTTGT-3’ | 371 | 58°C | GoTaq PCR Core System I |
| PTH1R_Ex9_M13_F  PTH1R_Ex9_M13_R | 9 | Fw  Rev | 37  36 | 60.10  60.25 | 5’-tgtaaaacgacggccagtAACCCAGCTTCCTGTCCAC-3’  5’-caggaaacagctatgaccACGGGTTTGAGTGGCTGA-3’ | 435 | 58°C | GC-rich PCR System |
| PTH1R_Ex10_M13_F  PTH1R_Ex10_M13_R | 10 | Fw  Rev | 35  36 | 58.84  60.24 | 5'-tgtaaaacgacggccagtAGCCCAGCCCTGACTTC-3'  5'-caggaaacagctatgaccCTCTCCCTGTCACCCACG-3' | 315 | 58°C | GoTaq PCR Core System I |
| PTH1R_Ex11 12_M13_F  PTH1R_Ex11 12_M13_R | 11+12 | Fw  Rev | 37  38 | 60.17  59.70 | 5'-tgtaaaacgacggccagtGGGGAATGACCTTGTGGAC-3'  5'-caggaaacagctatgaccAAGCCTCCTAGGTCCCTGTC-3' | 644 | 58°C | GoTaq PCR Core System I |
| PTH1R_Ex13_M13_F  PTH1R_Ex13_M13_R | 13 | Fw  Rev | 38  38 | 59.14  60.38 | 5'-tgtaaaacgacggccagtTGACAGAGCAGAGCCTATGG-3'  5'-caggaaacagctatgaccCATGGTACCAGGACTCAGGG-3' | 282 | 58°C | GoTaq PCR Core System I |
| PTH1R_Ex14 15_M13_F  PTH1R_Ex14 15_M13_R | 14+15 | Fw  Rev | 38  38 | 59.17  60.71 | 5'-tgtaaaacgacggccagtAATGCTTGTTGAAGGGGAAG-3'  5'-caggaaacagctatgaccAAATGAGCCTTGAGGAAGCC-3' | 428 | 58°C | GoTaq PCR Core System I |
| PTH1R_Ex16a_M13_F  PTH1R_Ex16a_M13_R | 16a | Fw  Rev | 35  35 | 61.76  60.08 | 5'-tgtaaaacgacggccagtTTCCGTGCTGGGTGTCC-3'  5'-caggaaacagctatgaccAGCAGGAGCCGTTGAGG-3' | 431 | 57°C | GoTaq PCR Core System I |
| PTH1R_Ex16b_M13_F  PTH1R_Ex16b_M13_R | 16b | Fw  Rev | 35  36 | 59.45  60.35 | 5'-tgtaaaacgacggccagtCCACTGCCACCACCAAC-3'  5'-caggaaacagctatgaccACAGCTTTCCCTGGCTCC-3' | 466 | 57°C | GoTaq PCR Core System I |
